# SCLEREDEMA DIABETICORUM WITH UNUSUAL PRESENTATION AND FATAL OUTCOME

**DOI:** 10.4103/0019-5154.44798

**Published:** 2008

**Authors:** Francisco J Ruiz Miyares, Renju Kuriakose, Dirk T Deleu, Naghi Abd El-Wahad, Hassan Al-Hail

**Affiliations:** *From Department of Neurology, Hamad General Hospital, Doha, State of Qatar*; 1 *From Department of Radiology, Hamad General Hospital, Doha, State of Qatar*

**Keywords:** *Atypical*, *neuropathy*, *restrictive*, *scleredema diabeticorum*, *fatal outcome*, *unusual presentation*

## Abstract

We present a case of Scleredema Diabeticorum (SD) in a patient with diabetic neuropathy and restrictive respiratory disease with unusual skin lesion distribution. The onset of dermatologic symptoms heralded a progressive respiratory disease with constrictive component. Painful diabetic neuropathy was noteworthy and difficult to relieve. Predominantly, distribution of the skin lesions on the thighs makes the case exceptional. T2-weighted MRI showed abnormal hyperintensities along the muscles of the thighs in correspondence with the skin lesions. Gait and respiratory symptoms progressively worsened. After a transient remitting period, he developed sudden shortening of breath, arrested and expired at home.The atypical distribution of the skin lesions with further involvement of underlying muscles plus concomitant polyneuropathy and respiratory constrictive disease with sudden death is quite unusual and aggressive presentation of SD.

## Introduction

Scleredema diabeticorum (SD) can be considered as a major dermatological complication of Diabetes Mellitus (DM). Histologically, there is a remarkable deposition of mucin along the deep dermis layers.^1^–^3^

The typical skin lesions are ill-defined, nonpitting, erythematous, indurated plaques, with "peau d’orange“ appearence and its distribution is mainly in the upper part of the body – in the neck, trunk and upper limbs. Face is frequently involved and hinders emotional expression and proper utterance of words.

The distribution in lower limbs is rare, and symmetric lesions in both thighs are, therefore, exceptional.^2^

The pathogenesis is not known, although the increased expression of collagen producing fibroblasts in the skin of affected individuals has been demonstrated.^3^ Diverse infectious conditions, i.e., upper respiratory tract infections, influenza, mumps, measles, pertussis, diphtheria, and cytomegalovirus can precede the dermatologic lesions.

Dysimmune processes can also be related with SD. Monoclonal gammopathy, multiple myeloma and other plasma cell dyscrasias have been associated with SD; however, the intrinsic mechanism is not known.^4^

Rarely, post-traumatic cases of SD have been described in association with trophic skin changes due to micro-angiopathy and sensory neuropathy that are common in DM and might facilitate mechanic, repetitive, damage.^5^

Long-term DM, mainly in those cases with poor control, is one of the outstanding associated conditions for SD.

The influence of SD lesions on other organs and tissues has been evidenced mainly through cardiovascular, respiratory and digestive dysfunctions. Cases of cardiac involvement ranging from transient arrhythmias, constrictive pericarditis, cardiomyopathies to sudden death have been reported.^6^ Respiratory complications of SD are not exceptional and range from persistent upper tract infections to severe constrictive lung disease.^7^

Dysphagia and constipation can be difficult to manage and excess of collagen fibers have been observed in bowel biopsies.^8^

The presentation of SD thigh lesions symmetrically with progressive constrictive respiratory disease with fatal outcomes has not been described up to our knowledge.

## Case Report

A 55-year-old male with 10-year history of diabetes mellitus type II. After 2 years, the patient noticed skin lesions that grew relentlessly in both thighs and legs. They were nonpitting erythematous, ill-delimitated with pronounced thickening and peau d’orange appearance. Three years prior to these events, the patient had numbness and pain in both legs, mainly distally, that worsened after the onset of skin lesions and walking was almost impossible without assistance. At the same time, he experienced frequent shortening of breath with nonproductive coughing and he was admitted several times.

**Fig. 1:**
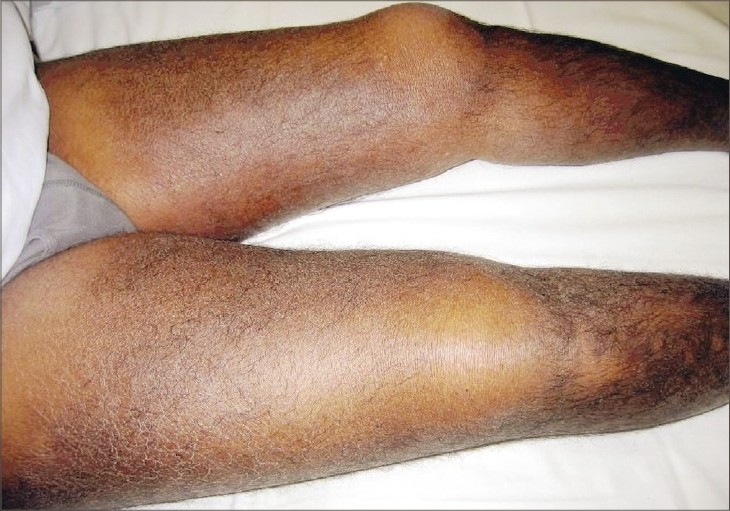
Corrugated skin in both thighs with erythematous induration and hyperpigmentation

On examination, the patient had moderate decreased sensory (touch and pain and in a less extent, position and vibration) with stock and glove distribution, symmetrical, as well as a moderate, generalized tendon hyporreflexia (2 minus). Skin lesions were prominent in both superior and anterior aspect of both thighs with ill-defined limits, erythematous, hyperpigmented and corrugated skin, typically peau d’orange (Fig. 1). Skin biopsy revealed the ballooning and swelling of collagen bundles with few scattered lymphocytes. Protein electrophoresis showed increased gamma globulin polyclonal bands.

**Fig. 2:**
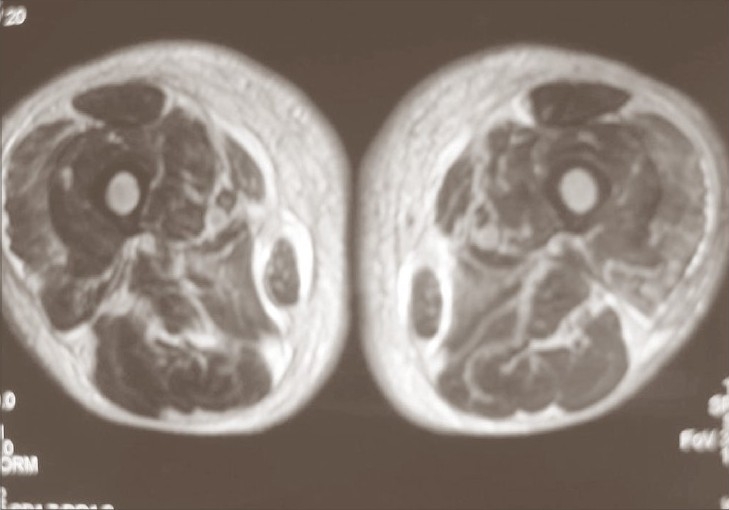
T2-weighted MRI with fat suppression technique shows thickening of the skin and edema with hyperintense signal that involves the muscles of both thighs, predominantly the left one

MRI of thighs revealed diffuse thickening of the skin with subcutaneous edema surrounding the muscles and entering the intermuscular compartment (Fig. 2).

After a period of few weeks, in addition to the constrictive respiratory disease, the patient developed congestive heart failure. At home, the patient had sudden dyspnea, arrested and died in spite of resuscitation maneuvers.

## Discussion

Sudden death in scleredema diabeticorum is exceptional and might be related with underlying involvement of heart and lungs.^7^ Skin lesions predominance in both thighs is atypical. MRI showed an increased signal in T2 that involved deeper structures, including fascia and muscles, that are indicative of active process and consistent with myositis – a rare association with SD.^9^

Other conditions such as blood dyscrasias can negatively influence the outcomes and serum protein electrophoresis should be systematically performed to rule out monoclonal gammopathy or paraproteinemia that often coexist in these patients. Our case showed increased gamma globulin polyclonal bands of unspecific chronic inflammatory response.

In the differentials, systemic sclerosis (SSc), amyloidosis and dermatomyositis should be carefully considered. In SSc, Raynaud's phenomenon and abnormalities of ungual capillaries are excluded, in view that this is a predominantly vasculitic condition with internal organ involvement.^10^ Amyloidosis has distinctive ecchymoses or waxy papules mainly in a facial distribution pattern.^11^ Dermatomyositis is a combination of muscle weakness and heliotrope rash and hyperpigmentation with a photosensitive distribution.^12^

SD is a dermatologic complication of long term DM that is usually considered to be benign; most of the times, it is not modified with metabolic control, thereby ensuing relentless deterioration.

Cardiopulmonary dysfunction in SD with atypical features can be considered as cause-related more than a random association in the absence of concomitant malignancy. The severity of the skin lesions with involvement of underlying muscles, respiratory manifestations and sudden demise of the patient under study support the hypothesis of a severe systemic disease that can end fatally, regardless of careful assessment and rational management.
